# Immunogenicity of Ad26.COV2.S vaccine against SARS-CoV-2 variants in humans

**DOI:** 10.1038/s41586-021-03681-2

**Published:** 2021-06-09

**Authors:** Galit Alter, Jingyou Yu, Jinyan Liu, Abishek Chandrashekar, Erica N. Borducchi, Lisa H. Tostanoski, Katherine McMahan, Catherine Jacob-Dolan, David R. Martinez, Aiquan Chang, Tochi Anioke, Michelle Lifton, Joseph Nkolola, Kathryn E. Stephenson, Caroline Atyeo, Sally Shin, Paul Fields, Ian Kaplan, Harlan Robins, Fatima Amanat, Florian Krammer, Ralph S. Baric, Mathieu Le Gars, Jerald Sadoff, Anne Marit de Groot, Dirk Heerwegh, Frank Struyf, Macaya Douoguih, Johan van Hoof, Hanneke Schuitemaker, Dan H. Barouch

**Affiliations:** 1 Center for Virology and Vaccine Research, Beth Israel Deaconess Medical Center, Boston, MA USA; 2 Ragon Institute of MGH, MIT and Harvard, Cambridge, MA USA; 3 Harvard Medical School, Boston, MA USA; 4 University of North Carolina at Chapel Hill, Chapel Hill, NC USA; 5 Adaptive Biotechnologies, Seattle, WA USA; 6 Icahn School of Medicine at Mount Sinai, New York, NY USA; 7 Janssen Vaccines & Prevention, Leiden, The Netherlands; 8 Janssen Research & Development, Beerse, Belgium

**Keywords:** Vaccines, SARS-CoV-2

## Abstract

The Ad26.COV2.S vaccine^1–3^ has demonstrated clinical efficacy against symptomatic COVID-19, including against the B.1.351 variant that is partially resistant to neutralizing antibodies^1^. However, the immunogenicity of this vaccine in humans against SARS-CoV-2 variants of concern remains unclear. Here we report humoral and cellular immune responses from 20 Ad26.COV2.S vaccinated individuals from the COV1001 phase I–IIa clinical trial^2^ against the original SARS-CoV-2 strain WA1/2020 as well as against the B.1.1.7, CAL.20C, P.1 and B.1.351 variants of concern. Ad26.COV2.S induced median pseudovirus neutralizing antibody titres that were 5.0-fold and 3.3-fold lower against the B.1.351 and P.1 variants, respectively, as compared with WA1/2020 on day 71 after vaccination. Median binding antibody titres were 2.9-fold and 2.7-fold lower against the B.1.351 and P.1 variants, respectively, as compared with WA1/2020. Antibody-dependent cellular phagocytosis, complement deposition and natural killer cell activation responses were largely preserved against the B.1.351 variant. CD8 and CD4 T cell responses, including central and effector memory responses, were comparable among the WA1/2020, B.1.1.7, B.1.351, P.1 and CAL.20C variants. These data show that neutralizing antibody responses induced by Ad26.COV2.S were reduced against the B.1.351 and P.1 variants, but functional non-neutralizing antibody responses and T cell responses were largely preserved against SARS-CoV-2 variants. These findings have implications for vaccine protection against SARS-CoV-2 variants of concern.

## Abstract (web summary)

Analysis of the immunogenicity of the Ad26.COV2.S vaccine against the B1.351 and P.1 SARS-CoV-2 variants of concern shows reduced neutralization antibody titres, but comparable T cell responses and antibody-dependent effector functions.

## Main

SARS-CoV-2 variants that partially escape from neutralizing antibodies to the WA1/2020 strain have emerged, including the B.1.351 variant that was first identified in South Africa^4,5^. Such variants of concern may reduce the efficacy of current vaccines and natural immunity from SARS-COV-2 strains that were prevalent at the beginning of the pandemic. The mRNA-1273 and BNT162b2 vaccines have been reported to induce lower neutralizing antibody titres against the B.1.351 variant than against the original WA1/2020 strain^4,6,7^. Additional SARS-CoV-2 variants include the B.1.1.7 variant that was first identified in the UK^8^, the P.1 and P.2 variants that were first identified in Brazil^9^, and the CAL.20C variant that was first identified in California^10^.

Ad26.COV2.S is a replication-incompetent human adenovirus type 26 (Ad26) vector^11^ that expresses a pre-fusion stabilized SARS-CoV-2 spike protein^12^ from the Wuhan 2019 strain, which is identical to the spike protein in the WA1/2020 strain. Ad26.COV2.S demonstrated protective efficacy against SARS-CoV-2 challenges in hamsters and non-human primates^3,13^ and showed safety and immunogenicity in humans^2,14^. In the phase III ENSEMBLE trial, a single dose of 5 × 10^10^ viral particles of Ad26.COV2.S resulted in 72%, 68% and 64% protection against moderate to severe COVID-19, and 86%, 88% and 82% protection against severe or critical COVID-19 in the US, Brazil and South Africa, respectively, by day 28 after vaccination^1^. In this trial, 69% of sequenced viruses from confirmed COVID-19 cases in Brazil were the P.2 variant, and 95% of sequenced viruses from confirmed COVID-19 cases in South Africa were the B.1.351 variant, which demonstrates that Ad26.COV2.S provided robust protective efficacy against these SARS-CoV-2 variants.

COV1001 is a multicentre, randomized, double-blind, placebo-controlled phase I–IIa trial to evaluate safety, reactogenicity and immunogenicity of Ad26.COV2.S at 5 × 10^10^ or 1 × 10^11^ viral particles administered intramuscularly as single-shot or two-shot vaccine schedules, 56 days apart, in healthy adults (NCT04436276)^14^. Cohort 1b enrolled 25 adults 18–55 years of age from 29 July 2020 to 7 August 2020 at a single site at Beth Israel Deaconess Medical Center (BIDMC), Boston, Massachusetts, for exploratory immunogenicity studies^2^. The study was approved by the BIDMC Institutional Review Board, and all participants provided written informed consent. Participants were randomly allocated to one of five experimental groups (*n* = 5 per group): (1) 5 × 10^10^ viral particles of Ad26.COV2.S on days 1 and 57 (low-dose–low-dose); (2) 5 × 10^10^ viral particles of Ad26.COV2.S on day 1 and placebo on day 57 as a single-shot vaccine (low-dose–placebo); (3) 1 × 10^11^ viral particles of Ad26.COV2.S on days 1 and 57 (high-dose–high-dose); (4) 1 × 10^11^ viral particles of Ad26.COV2.S on day 1 and placebo on day 57 as a single-shot vaccine (high-dose–placebo); or (5) placebo on days 1 and 57 (placebo–placebo).

## Antibody responses to variants

**Fig. 1:**
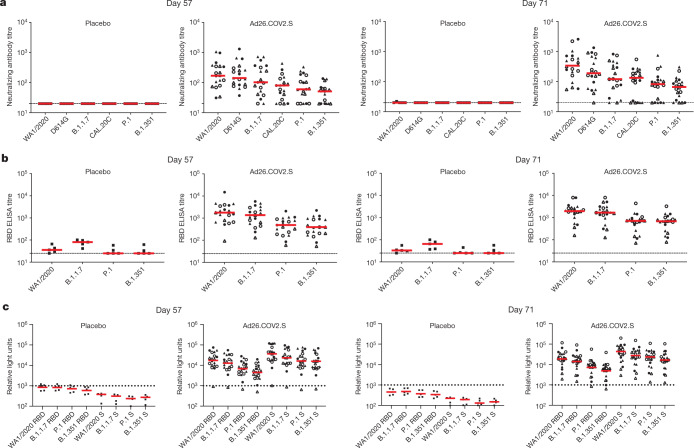
Neutralizing and binding antibody responses to SARS-CoV-2 variants. **a**, SARS-CoV-2 psVNA responses against WA1/2020, D614G, B.1.1.7, CAL.20C, P.1 and B.1.351 (**a**), RBD-specific binding antibodies by ELISA against WA1/2020, B.1.1.7, P.1 and B.1.351 (**b**) and RBD-specific and spike (S)-specific binding antibodies by ECLA against WA1/2020, B.1.1.7, P.1 and B.1.351 (Meso Scale Discovery panel 7) (**c**) on days 57 and 71. Red bars reflect median responses. Dotted lines reflect the lower limits of quantification. Filled squares denote placebo–placebo; filled circles denote high dose–placebo; open circles denote high dose–high dose; filled triangles denote low dose–placebo; and open triangles denote low dose–low dose. *n* = 25 independent samples (5 placebo recipients, 20 Ad26.COV2.S vaccine recipients).

**Extended Data Fig. 1:**
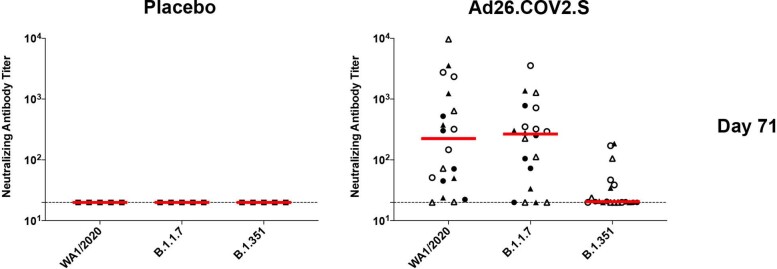
Live virus neutralizing antibody responses to SARS-CoV-2 variants. SARS-CoV-2 live virus neutralizing antibody responses against WA1/2020, B.1.1.7 and B.1.351. Red bars reflect median responses. Dotted lines reflect lower limits of quantitation. Filled squares, placebo–placebo; filled circles, high dose–placebo; open circles, high dose–high dose; filled triangles, low dose–placebo; open triangles, low dose–low dose. *n* = 25 independent samples (5 placebo recipients, 20 Ad26.COV2.S vaccine recipients).

Antibody responses were assessed against the SARS-CoV-2 WA1/2020 strain as well as against B.1.351 and other variants of concern. Using a luciferase-based pseudovirus neutralizing antibody (psVNA) assay^3,15,16^, the median psVNA titres were 169, 142, 102, 80, 60 and 51 against the WA1/2020, D614G, B.1.1.7, CAL.20C, P.1 and B.1.351 strains, respectively, on day 57 (Fig. 1a). The median psVNA titres were 340, 190, 121, 133, 102 and 67, respectively, against these variants on day 71. These data show a 3.3-fold reduction of psVNA titres against P.1 and a 5.0-fold reduction of psVNA titres against B.1.351 as compared with WA1/2020 on day 71. No psVNA titres were observed in placebo recipients. Live virus neutralizing antibody assays^17^ showed a greater than 10.6-fold reduction in antibody titres against B.1.351 as than against WA1/2020 on day 71 (Extended Data Fig. 1). This study was not powered to compare responses for the different vaccine doses or regimens.

On day 57, median receptor binding domain (RBD)-specific binding antibody enzyme-linked immunosorbent assay (ELISA) titres were 1,772, 1,364, 486 and 392 against the WA1/2020, B.1.1.7, P.1 and B.1.351 variants, respectively (Fig. 1b). On day 71, median ELISA titres were 1,962, 1,682, 714 and 683, respectively, against these variants. These data show a 1.2-, 2.7- and 2.9-fold reduction of ELISA titres against B.1.1.7, P.1 and B.1.351 RBD, respectively, as compared with WA1/2020 RBD on day 71. Minimal ELISA titres were observed in recipients that received the placebo.

An electrochemiluminescence assay (ECLA)^18^ was also used to evaluate spike- and RBD-specific binding antibody responses to WA1/2020, B.1.1.7, P.1 and B.1.351 (Fig. 1c). Similar to the ELISA titres, median RBD-specific ECLA responses against B.1.1.7, P.1 and B.1.351 were reduced 1.3-, 1.8- and 2.9-fold, and median spike-specific ECLA responses were reduced 1.6-, 1.8- and 2.6-fold, respectively, as compared with WA1/2020 on day 71.

**Fig. 2:**
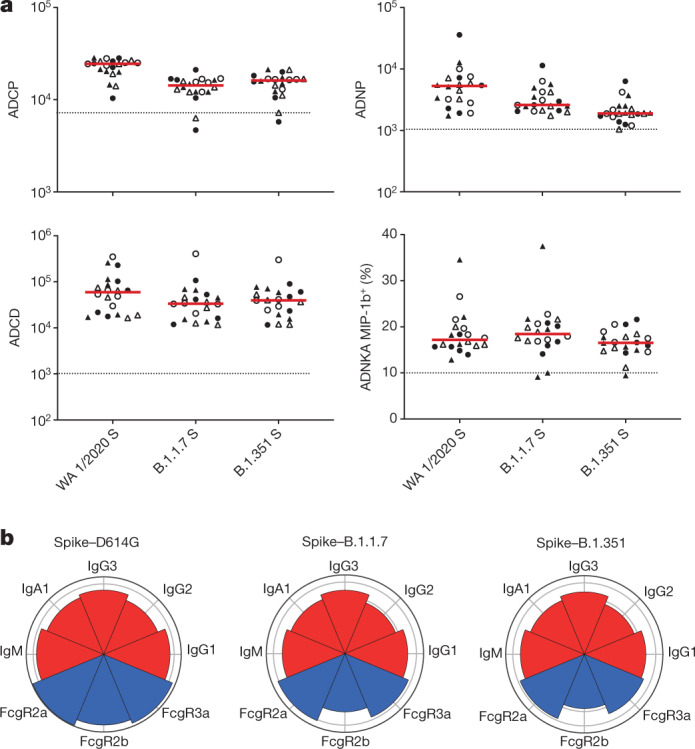
Systems serology to SARS-CoV-2 variants. **a**, Spike-specific ADCP, ADNP, ADCD and ADNKA responses against WA1/2020 (D614G), B.1.1.7 and B.1.351 on day 71. Red bars reflect median responses. Dotted lines reflect median of placebo recipients. Filled circles denote high dose–placebo; open circles denote high dose–high dose; filled triangles denote low dose–placebo; and open triangles denote low dose–low dose. **b**, Nightingale plots show the median levels of WA1/2020 (D614G), B.1.1.7, B.1.351 spike-specific isotype (IgM, IgA, IgG1, IgG2 and IgG3) (red) and FcγR2a, FcγR2b and FcγR3a (blue) binding. *n* = 20 independent samples from Ad26.COV2.S vaccine recipients.

**Extended Data Fig. 2:**
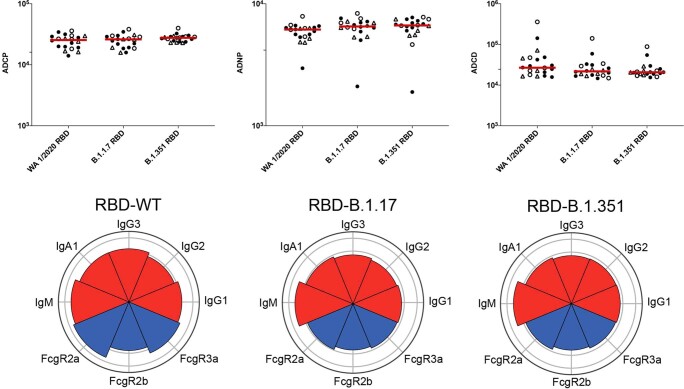
RBD-specific functional antibody responses to SARS-CoV-2 variants. Top, RBD-specific ADCP, ADNP and ADCD against WA1/2020 (D614G), B.1.1.7 and B.1.351. LD, low dose; HD, high dose; PL, placebo on day 71. Filled circles, high dose–placebo; open circles, high dose–high dose; filled triangles, low dose–placebo; open triangles, low dose–low dose. Bottom, RBD-specific isotype (IgG1, IgG3, IgA, IgM) (red) and FcγR2a, FcγR2b, FcγR3a (blue) binding against WA1/2020 (D614G), B.1.1.7, B.1.351 on day 71. *n* = 20 independent samples from Ad26.COV2.S vaccine recipients.

Antibody Fc effector function was assessed on day 71 by systems serology^19^, including antibody-dependent cellular phagocytosis (ADCP), antibody-dependent neutrophil phagocytosis (ADNP), antibody-dependent complement deposition (ADCD), and antibody-dependent natural killer cell activation (ADNKA). Spike-specific ADCP, ADNP, ADCD and ADNKA responses against the B.1.351 variant were 1.5-, 2.9-, 1.6- and 1.1-fold reduced, respectively, compared with the WA1/2020 strain with the D614G mutation (Fig. 2a). Comparable IgG, IgM and IgA subclasses and Fc-receptor binding were observed across the variants, with only a slight loss in FcγR2b binding compared to the WA1/2020 strain (Fig. 2b). RBD-specific ADCP, ADNP and ADCD responses were comparable against the WA1/2020, B.1.1.7 and B.1.351 variants (Extended Data Fig. 2). These data show robust spike- and RBD-specific Fc-effector functions against these SARS-CoV-2 variants.

## Cellular immune responses to variants

**Fig. 3:**
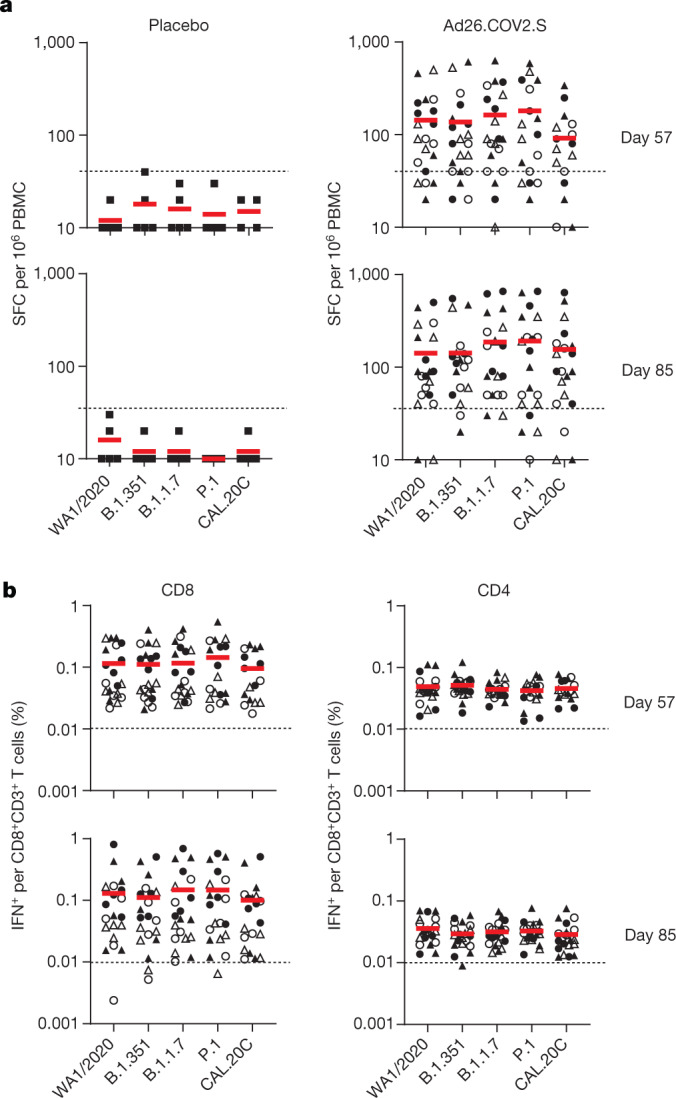
Cellular immune responses to SARS-CoV-2 variants. **a**, Spike-specific pooled peptide IFNγ ELISPOT responses against WA1/2020, B.1.351, B.1.1.7, P.1 and CAL.20C. *n* = 25 independent samples (5 placebo recipients, 20 Ad26.COV2.S vaccine recipients). PBMC, peripheral blood mononuclear cells. **b**, Spike-specific pooled peptide IFNγ CD4^+^ and CD8^+^ T cell responses by ICS assays against WA1/2020, B.1.351, B.1.1.7, P.1 and CAL.20C. SFC, spot-forming cells. Responses are shown on days 57 and 85. Red bars reflect median responses. Dotted lines reflect lower limits of quantification. Filled squares denote placebo–placebo; filled circles denote high dose–placebo; open circles denote high dose–high dose; filled triangles denote low dose–placebo; and open triangles denote low dose–low dose. *n* = 20 independent samples from Ad26.COV2.S vaccine recipients.

**Extended Data Fig. 3:**
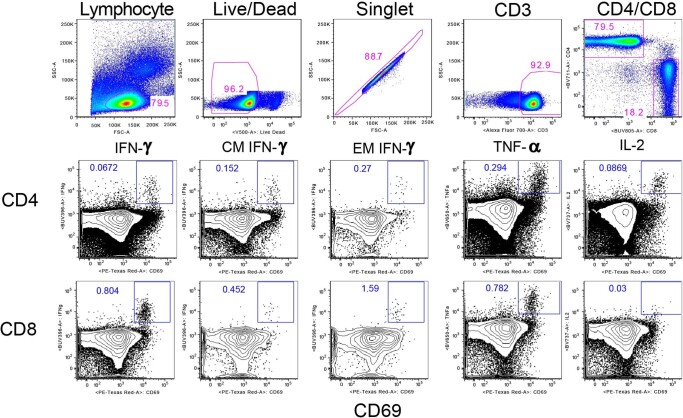
Representative gating for ICS assays. Sample gating plots are shown.

**Extended Data Fig. 4:**
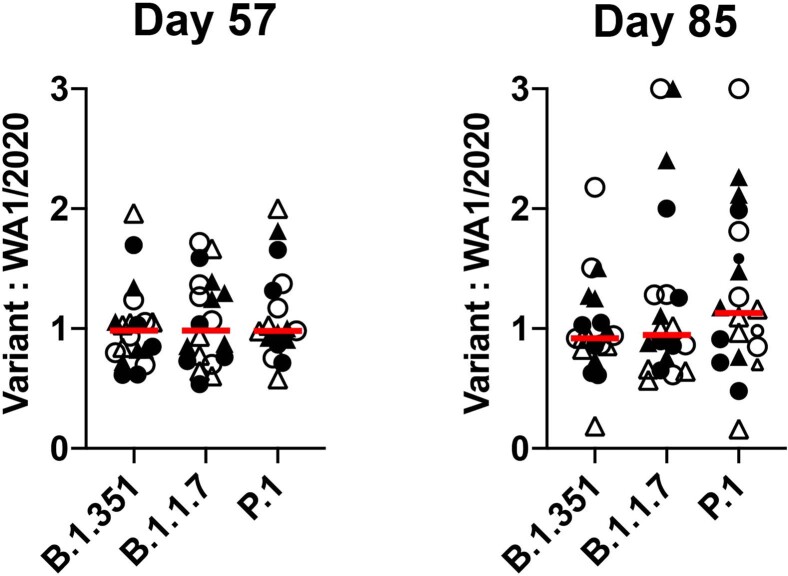
Ratio of variants versus WA1/2020 CD8^+^ T cell responses. Ratio of spike-specific pooled peptide IFNγ CD8^+^ T cell responses by ICS assays against B.1.351, B.1.1.7 and P.1 versus WA1/2020 on days 57 and 85. Red bars reflect median responses. Filled circles, high dose–placebo; open circles, high dose–high dose; filled triangles, low dose–placebo; open triangles, low dose–low dose. *n* = 20 independent samples from Ad26.COV2.S vaccine recipients.

**Extended Data Fig. 5:**
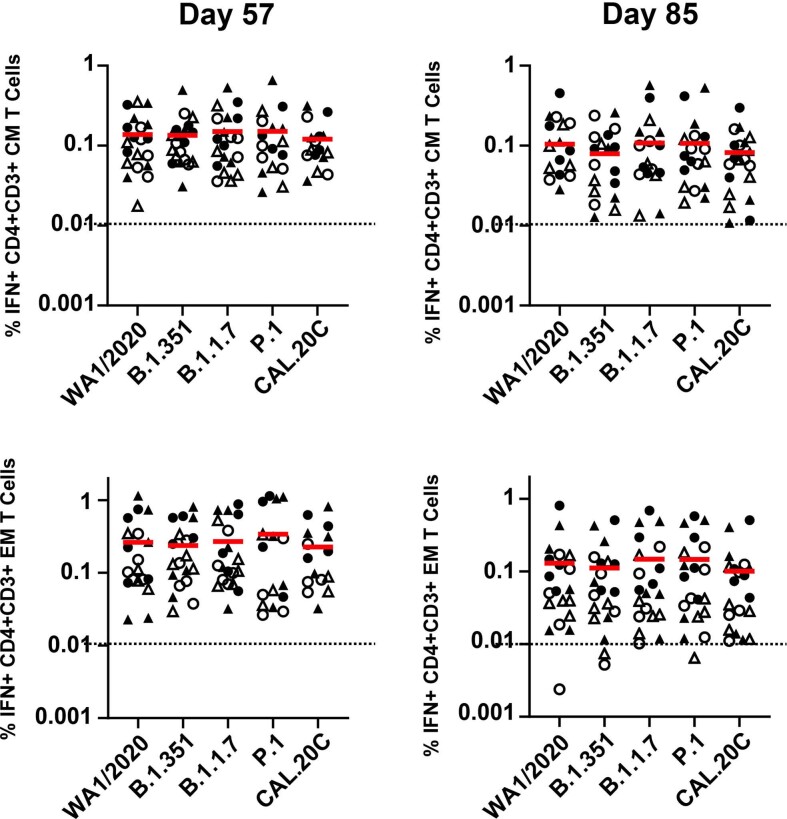
Central and effector memory CD8^+^ T cell responses to SARS-CoV-2 variants. Spike-specific pooled peptide IFNγ central memory CD27^+^CD45RA^−^ and effector memory CD27^−^CD45RA^−^ CD8^+^ T cell responses by ICS assays against WA1/2020, B.1.351, B.1.1.7, P.1 and CAL.20C on days 57 and 85. Red bars reflect median responses. Dotted lines reflect lower limits of quantification. Filled circles, high dose–placebo; open circles, high dose–high dose; filled triangles, low dose–placebo; open triangles, low dose–low dose. *n* = 20 independent samples from Ad26.COV2.S vaccine recipients.

**Extended Data Fig. 6:**
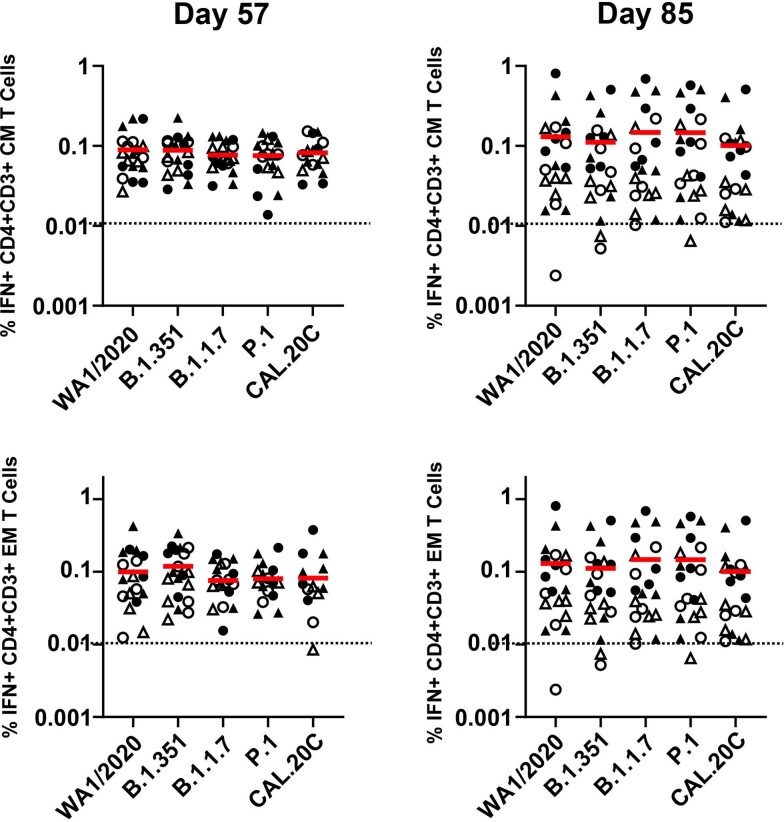
Central and effector memory CD4^+^ T cell responses to SARS-CoV-2 variants. Spike-specific pooled peptide IFNγ central memory CD27^+^CD45RA^−^ and effector memory CD27^−^CD45RA^−^ CD4^+^ T cell responses by ICS assays against WA1/2020, B.1.351, B.1.1.7, P.1 and CAL.20C on days 57 and 85. Red bars reflect median responses. Dotted lines reflect lower limits of quantification. Filled circles, high dose–placebo; open circles, high dose–high dose; filled triangles, low dose–placebo; open triangles, low dose–low dose. *n* = 20 independent samples from Ad26.COV2.S vaccine recipients.

**Extended Data Fig. 7:**
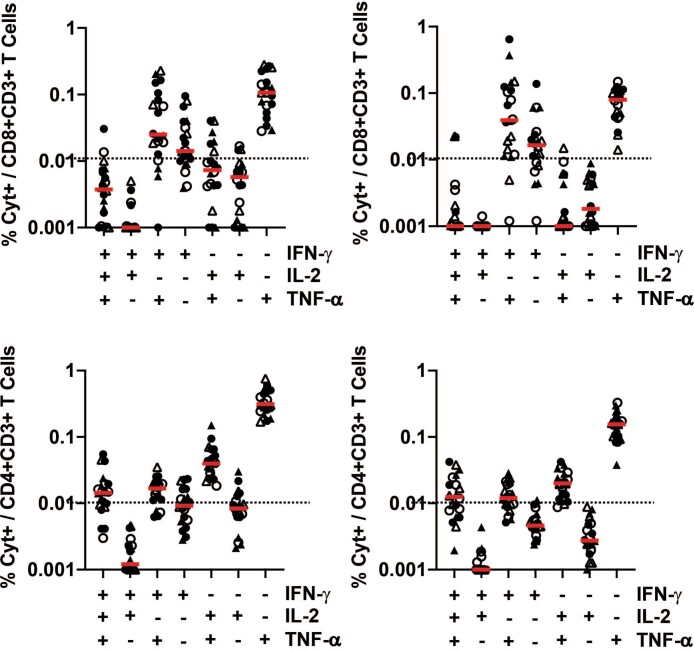
Polyfunctional CD8^+^ and CD4^+^ T cell responses. WA1/2020 spike-specific pooled peptide monofunctional and multifunctional IFNγ, IL-2 and TNF CD8^+^ and CD4^+^ T cell responses by ICS assays on days 57 and 85. Red bars reflect median responses. Dotted lines reflect lower limits of quantification. Filled circles, high dose–placebo; open circles, high dose–high dose; filled triangles, low dose–placebo; open triangles, low dose–low dose. *n* = 20 independent samples from Ad26.COV2.S vaccine recipients.

Spike-specific cellular immune responses were assessed by pooled peptide ELISPOT assays in peripheral blood mononuclear cells on days 57 and 85. IFNγ ELISPOT responses were comparable to WA1/2020, B.1.351, B.1.1.7, P.1 and CAL.20C at both time points, with no evidence of decreased responses against the variants (Fig. 3a). No spike-specific ELISPOT responses were observed in vaccine recipients who received placebo. Spike-specific CD8^+^ and CD4^+^ T cell responses were evaluated by multiparameter intracellular cytokine staining (ICS) assays on days 57 and 85 (Extended Data Fig. 3). IFNγ CD8^+^ and CD4^+^ T cell responses were comparable to WA1/2020, B.1.351, B.1.1.7, P.1 and CAL.20C variants (Fig. 3b). The median ratios of B.1.351, B.1.1.7 and P.1 to WA1/2020 IFNγ CD8^+^ T cell responses were 0.98, 0.98 and 0.98, respectively, on day 57, and 0.92, 0.94 and 1.26, respectively, on day 85 (Extended Data Fig. 4). Central memory CD27^+^CD45RA^−^ and effector memory CD27^−^CD45RA^−^ CD4^+^ and CD8^+^ T cell responses were also comparable across these variants (Extended Data Figs. 5, 6). These data show that spike-specific cellular immune responses were not detectably affected by SARS-CoV-2 variants. Polyfunctional analyses showed that CD8^+^ T cells were primarily IFNγ, TNF and both IFNγ and TNF responses, whereas CD4^+^ T cells were primarily TNF; IFNγ and TNF; IL-2 and TNF; and IFNγ, IL-2 and TNF responses (Extended Data Fig. 7).

**Fig. 4:**
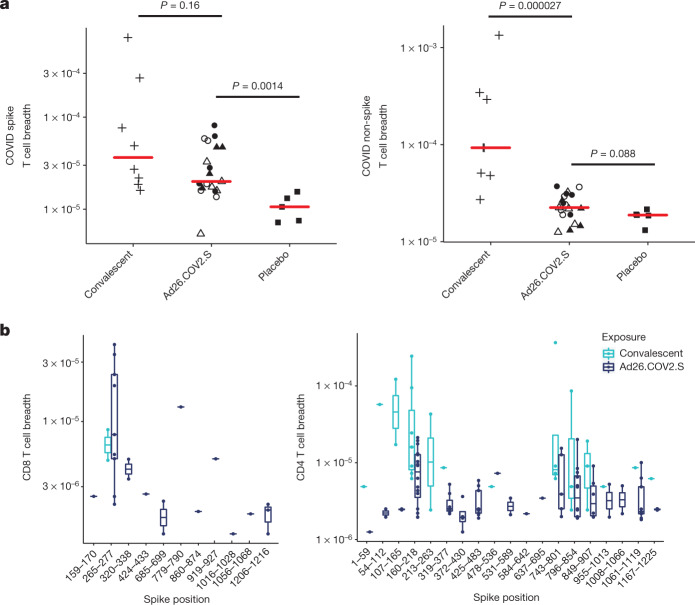
TCRβ repertoire analysis. **a**, Spike and non-spike T cell breadth by TCRβ sequencing on day 63. *P* values were determined by two-sided Wilcoxon rank-sum tests. Red bars reflect median responses. **b**, Breadth of spike-specific CD8^+^ and CD4^+^ T cell responses. Filled squares denote placebo–placebo; filled circles denote high dose–placebo; open circles denote high dose–high dose; filled triangles denote low dose–placebo; open triangles denote low dose–low dose; and plus signs denote convalescent samples. In the box-and-whisker plots, the middle line reflects the median, the box reflects the 25th–75th percentiles and the whiskers extend the full range up to 1.5× the interquartile range, with outlier points marked individually. *n* = 32 independent samples (8 SARS-CoV-2 convalescent individuals, 5 placebo recipients, 19 Ad26.COV2.S vaccine recipients).

**Extended Data Fig. 8:**
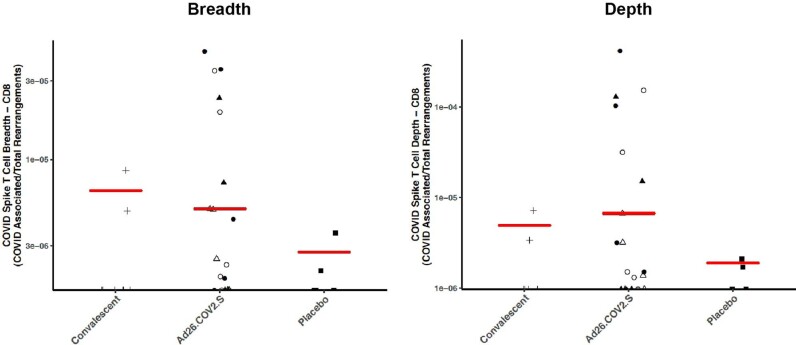
CD8^+^ TCRβ repertoire analysis. CD8^+^ T cell breadth and depth by TCRβ sequencing on day 57. Red bars reflect median responses. Filled squares, placebo–placebo; filled circles, high dose–placebo; open circles, high dose–high dose; filled triangles, low dose–placebo; open triangles, low dose–low dose; plus signs, convalescent samples. *n* = 32 independent samples (8 SARS-CoV-2 convalescent individuals, 5 placebo recipients, 19 Ad26.COV2.S vaccine recipients).

**Extended Data Fig. 9:**
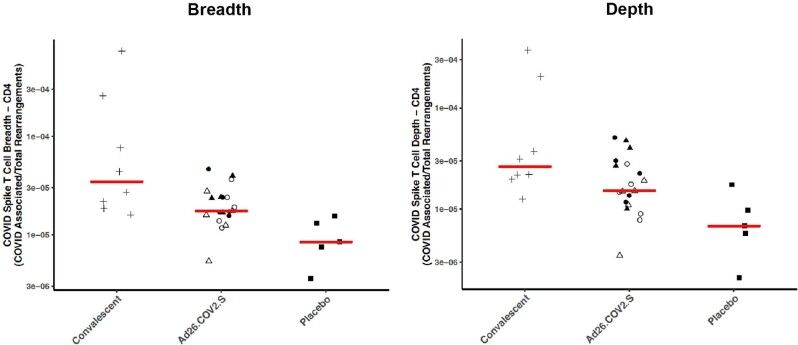
CD4^+^ TCRβ repertoire analysis. CD4^+^ T cell breadth and depth by TCRβ sequencing on day 57. Red bars reflect median responses. Filled squares, placebo–placebo; filled circles, high dose–placebo; open circles, high dose–high dose; filled triangles, low dose–placebo; open triangles, low dose–low dose; plus signs, convalescent samples. *n* = 32 independent samples (8 SARS-CoV-2 convalescent individuals, 5 placebo recipients, 19 Ad26.COV2.S vaccine recipients).

To evaluate the specificity and breadth of individual T cell receptors (TCRs) after vaccination, TCRβ sequencing^20^ was performed to define the repertoires of 8 convalescent individuals and 19 participants receiving the vaccine and 5 receiving placebo on day 63 (Extended Data Table 1). To identify SARS-CoV-2 specific TCRs, the observed TCRs were compared to a TCR dataset that had previously been determined to be SARS-CoV-2-specific and enriched in subjects with natural infection relative to placebos^21^. The breadth (unique rearrangements) and depth (frequency of TCRs) of TCRs specific to either spike or non-spike SARS-CoV-2 proteins were determined, although these analyses may have underestimated total T cell responses. Higher breadth of spike-specific TCRs was observed in vaccine recipients compared with placebos (*P* = 0.0014, Wilcoxon rank-sum test) (Fig. 4a, Extended Data Figs. 8, 9). By contrast, the breadth of non-spike TCRs was comparable in vaccine recipients and controls, as expected because the vaccine did not contain any non-spike immunogens. Substantial breadth of CD8^+^ and CD4^+^ T cell responses was also observed (Fig. 4b).

## Discussion

SARS-CoV-2 variants have emerged with several mutations in targets of neutralizing antibodies, such as the E484K mutation. Median pseudovirus neutralizing antibody titres induced by Ad26.COV2.S were 5.0-fold lower against the B.1.351 variant and 3.3-fold lower against the P.1 variant as compared with the original WA1/2020 strain, which is a comparable reduction of psVNA titres that has been reported for other vaccines^4,6,7^. By contrast, functional non-neutralizing antibody responses and CD8^+^ and CD4^+^ T cell responses were largely preserved against SARS-CoV-2 variants of concern.

In the phase III ENSEMBLE trial^1^, Ad26.COV2.S was evaluated in the USA, Latin America including Brazil, and South Africa. In South Africa, 95% of sequenced viruses from COVID-19 cases were of the B.1.351 variant, and in Brazil, 69% of sequenced viruses from COVID-19 cases were of the P.2 lineage. Protective efficacy of Ad26.COV2.S against severe or critical disease was similar in all geographic locations by day 28, and protective efficacy against moderate to severe disease was only slightly reduced in South Africa compared with the USA. Although the mechanistic correlates of protection for COVID-19 are not yet known, the robust protective efficacy in these regions despite reduced neutralizing antibodies raises the possibility that functional non-neutralizing antibodies and/or CD8^+^ T cell responses may also contribute to protection. Indeed, TCRβ sequencing revealed substantial breadth of T cell responses in individuals vaccinated with Ad26.COV2.S. Alternatively, it is possible that low levels of neutralizing antibodies are sufficient for protection. In a non-human primate model, adoptive transfer of purified IgG was sufficient for protection against SARS-CoV-2 if titres of psVNA exceeded a threshold of approximately 50, but CD8^+^ T cells also contributed to protection if antibody titres were subprotective^22,23^.

In conclusion, neutralizing antibody responses elicited by Ad26.COV2.S were reduced against the B.1.351 and P.1 variants, but other functional antibody responses and T cell responses were largely preserved against these variants. The relevance of these immune parameters to mechanistic correlates of vaccine efficacy remains to be determined.

## Methods

### Data reporting

No statistical methods were used to predetermine sample size. The study was randomized, blinded, and placebo controlled. Investigators were blinded to allocation during experiments and outcome assessment.

### Pseudovirus-based neutralization assay

The SARS-CoV-2 pseudoviruses expressing a luciferase reporter gene were generated in an approach similar to that described previously^16,23^. In brief, the packaging plasmid psPAX2 (AIDS Resource and Reagent Program), luciferase reporter plasmid pLenti-CMV Puro-Luc (Addgene), and spike protein expressing pcDNA3.1-SARS CoV-2 SΔCT of variants were co-transfected into HEK293T cells (ATCC, mycoplasma tested) using lipofectamine 2000 (ThermoFisher). Pseudoviruses of SARS-CoV-2 variants were generated by using the WA1/2020 strain (Wuhan/WIV04/2019, GISAID accession ID: EPI_ISL_402124), D614G mutation, B.1.1.7 variant (GISAID accession ID: EPI_ISL_601443), CAL.20C (GISAID accession ID: EPI_ISL_824730), P.1 (GISAID accession ID: EPI_ISL_792683), or B.1.351 variant (GISAID accession ID: EPI_ISL_712096). The supernatants containing the pseudotype viruses were collected 48 h after transfection, and then were purified by centrifugation and filtration with a 0.45-µm filter. To determine the neutralization activity of the plasma or serum samples from participants, HEK293T-hACE2 cells were seeded in 96-well tissue culture plates at a density of 1.75 x 10^4^ cells/well overnight. Three-fold serial dilutions of heat-inactivated serum or plasma samples were prepared and mixed with 50 µl pseudovirus. The mixture was incubated at 37 ^o^C for 1 h before being added to HEK293T-hACE2 cells. Forty-eight hours after infection, cells were lysed in Steady-Glo Luciferase Assay (Promega) according to the manufacturer’s instructions. SARS-CoV-2 neutralization titres were defined as the sample dilution at which a 50% reduction in relative light unit (RLU) was observed relative to the average of the virus control wells.

### Live virus neutralization assay

Full-length SARS-CoV-2 WA1/2020, B.1.351 and B.1.1.7, viruses were designed to express nanoluciferase (nLuc) and were recovered via reverse genetics^17^. One day before the assay, Vero E6 USAMRID cells were plated at 20,000 cells per well in clear-bottom black-walled plates. Cells were inspected to ensure confluency on the day of assay. Serum samples were tested at a starting dilution of 1:20 and were serially diluted threefold up to nine dilution spots. Serially diluted serum samples were mixed in equal volume with diluted virus. Antibody–virus and virus-only mixtures were then incubated at 37 °C with 5% CO_2_ for one hour. After incubation, serially diluted sera and virus only controls were added in duplicate to the cells at 75 plaque-forming units at 37 °C with 5% CO_2_. Twenty-four hours later, the cells were lysed, and luciferase activity was measured via Nano-Glo Luciferase Assay System (Promega) according to the manufacturer specifications. Luminescence was measured by a Spectramax M3 plate reader (Molecular Devices). Virus neutralization titres were defined as the sample dilution at which a 50% reduction in RLU was observed relative to the average of the virus control wells.

### ELISA

WA1/2020, B.1.1.7 and B.1.351 RBD-specific binding antibodies were assessed by ELISA. In brief, 96-well plates were coated with 2 μg ml^−1^ RBD proteins (provided by F. Krammer) in 1× DPBS and incubated at 4 °C overnight. After incubation, plates were washed once with wash buffer (0.05% Tween 20 in 1× DPBS) and blocked with 350 μl casein block per well for 2–3 h at room temperature. After incubation, block solution was discarded and plates were blotted dry. Serial dilutions of heat-inactivated serum diluted in casein block were added to wells and plates were incubated for 1 h at room temperature, before three further washes and a 1 h incubation with a 1:4,000 dilution of anti-human IgG HRP (Invitrogen) at room temperature in the dark. Plates were then washed three times, and 100 μl of SeraCare KPL TMB SureBlue Start solution was added to each well; plate development was halted by the addition of 100 μl SeraCare KPL TMB Stop solution per well. The absorbance at 450 nm, with a reference at 650 nm, was recorded using a VersaMax microplate reader. For each sample, ELISA endpoint titre was calculated in Graphpad Prism software, using a four-parameter logistic curve fit to calculate the reciprocal serum dilution that yields a corrected absorbance value (450–650 nm) of 0.2. The log_10_-transformed endpoint titres are reported.

### ECLA

ECLA plates (MesoScale Discovery SARS-CoV-2 IgG N05CA-1; panel 7) were designed and produced with up to nine antigen spots in each well. The antigens included were WA1/2020, B.1.1.7, P.1 and B.1.351 S and RBD. The plates were blocked with 50 μl of blocker A (1% BSA in MilliQ water) solution for at least 30 min at room temperature shaking at 700 rpm with a digital microplate shaker. During blocking, the serum was diluted 1:5,000. The plates were then washed three times with 150 μl of the MSD kit Wash Buffer, blotted dry, and 50 μl of the diluted samples were added in duplicate to the plates and set to shake at 700 rpm at room temperature for at least 2 h. The plates were again washed three times and 50 μl of SULFO-Tagged anti-Human IgG detection antibody diluted to 1× in Diluent 100 was added to each well and incubated shaking at 700 rpm at room temperature for at least 1 h. Plates were then washed three times and 150 μl of MSD GOLD Read Buffer B was added to each well and the plates were read immediately after on a MESO QuickPlex SQ 120 machine. MSD titres for each sample was reported as RLU, which were calculated as sample RLU minus the blank RLU for each spot for each sample. The limit of detection was defined as 1,000 RLU for each assay.

### Systems serology

Both the biophysical and functional quality of polyclonal vaccine induced SARS-CoV-2 antibodies were profiled using systems serology^19^. Biophysical profiling was performed using a custom Luminex based assay where individuals bar-coded beads were coated with spike (S) or (RBD) variants by carboxy coupling. The D614G, B.1.1.7 and B.1.351 variants (provided by E. Ollman Saphire and F. Krammer) were profiled. The overall levels of IgG1, IgG2, IgG3, IgA, IgM and FcγR2a, FcγR2b, FcγR3a and FcγR3b binding were assessed. Functional profiling included the assessment of ADCP, ADNP, ADCD and ADNKA. In brief, for the ADCP, ADNP and ADCD assays, fluorescent beads (LifeTechnologies) were coupled via carboxy-coupling, and plasma was added, allowing immune complex formation, excess antibodies were washed away, followed by the addition of THP1 monocytes, primary neutrophils, or guinea pig complement, individually, respectively. The level of phagocytosis and complement deposition was assessed by flow cytometry. For ADNKA, ELISA plates were coated with antigen, followed by the addition of plasma. Excess antibodies were washed away following by the addition of primary natural killer cells. Natural killer cells were treated with Golgi Stop (BD) and brefeldin A (Sigma Aldrich) and were stained for the surface markers CD56, CD16 and CD3 and for activity markers CD107a (BD) and MIP-1b (BD). Fluorescence was determined by flow cytometry. Natural killer cells were classified as CD56^+^CD16^+^CD3^−^.

### ELISPOT assay

ELISPOT plates were coated with mouse anti-human IFNγ monoclonal antibody from MabTech at 1 μg per well and incubated overnight at 4 °C. Plates were washed with DPBS, and blocked with R10 medium (RPMI with 10% heat-inactivated FBS with 1% of 100× penicillin–streptomycin, 1 M HEPES, 100 mM sodium pyruvate, 200 mM l-glutamine, and 0.1% of 55 mM 2-mercaptoethanol) for 2–4 h at 37 °C. SARS-CoV-2 pooled spike peptides from WA1/2020, B.1.351, B.1.1.7, P.1 and CAL.20C (21st Century Biochemicals) were prepared and plated at a concentration of 2 μg per well, and 100,000 cells per well were added to the plate. The peptides and cells were incubated for 15–20 h at 37 °C. All steps after this incubation were performed at room temperature. The plates were washed with ELISPOT wash buffer and incubated for 2–4 h with biotinylated mouse anti-human IFNγ monoclonal antibody from MabTech (1 μg ml^−1^). The plates were washed a second time and incubated for 2–3 h with conjugated Goat anti-biotin AP from Rockland (1.33 μg ml^−1^). The final wash was followed by the addition of Nitor-blue Tetrazolium Chloride/5-bromo-4-chloro 3′ indolyphosphate p-toludine salt (NBT/BCIP chromagen) substrate solution for 7 min. The chromagen was discarded and the plates were washed with water and dried in a dim place for 24 h. Plates were scanned and counted on a Cellular Technologies Limited Immunospot Analyzer.

### ICS assay

Peripheral blood mononuclear cells (10^6^ per well) were re-suspended in 100 μl of R10 medium supplemented with CD49d monoclonal antibody (1 μg ml^−1^) and CD28 monoclonal antibody (1 μg ml^−1^). Each sample was assessed with mock (100 μl of R10 plus 0.5% DMSO; background control), pooled S peptides from WA1/2020, B.1.351, B.1.1.7, P.1 and CAL.20C (21st Century Biochemicals) (2 μg ml^−1^), or 10 pg ml^−1^ phorbol myristate acetate and 1 μg ml^−1^ ionomycin (Sigma-Aldrich) (100 μl; positive control) and incubated at 37 °C for 1 h. After incubation, 0.25 μl of GolgiStop and 0.25 μl of GolgiPlug in 50 μl of R10 was added to each well and incubated at 37 °C for 8 h and then held at 4 °C overnight. The next day, the cells were washed twice with DPBS, stained with aqua live/dead dye for 10 min and then stained with predetermined titres of monoclonal antibodies against CD279 (clone EH12.1, BB700), CD4 (clone L200, BV711), CD27 (clone M-T271, BUV563), CD8 (clone SK1, BUV805), CD45RA (clone 5H9, APC H7) for 30 min. Cells were then washed twice with 2% FBS/DPBS buffer and incubated for 15 min with 200 μl of BD CytoFix/CytoPerm Fixation/Permeabilization solution. Cells were washed twice with 1× Perm Wash buffer (BD Perm/Wash Buffer 10× in the CytoFix/CytoPerm Fixation/Permeabilization kit diluted with MilliQ water and passed through 0.22-μm filter) and stained with intracellularly with monoclonal antibodies against Ki67 (clone B56, BB515), IL-21 (clone 3A3-N2.1, PE), CD69 (clone TP1.55.3, ECD), IL-10 (clone JES3-9D7, PE CY7), IL-13 (clone JES10-5A2, BV421), IL-4 (clone MP4-25D2, BV605), TNF (clone Mab11, BV650), IL-17 (clone N49-653, BV750), IFNγ (clone B27; BUV395), IL-2 (clone MQ1-17H12, BUV737), IL-6 (clone MQ2-13A5, APC), CD3 (clone SP34.2, Alexa 700), for 30 min. Cells were washed twice with 1× Perm Wash buffer and fixed with 250 μl of freshly prepared 1.5% formaldehyde. Fixed cells were transferred to 96-well round bottom plate and analysed by BD FACSymphony system. Data were analysed with FlowJo v.9.9.

### T cell receptor variable beta chain sequencing

Immunosequencing of the CDR3 regions of human TCRβ chains was performed using the immunoSEQ Assay (Adaptive Biotechnologies). Extracted genomic DNA was amplified in a bias-controlled multiplex PCR, followed by high-throughput sequencing. Sequences were collapsed and filtered to identify and quantitate the absolute abundance of each unique TCRβ CDR3 region for further analysis as previously described^20^. The fraction of T cells was calculated by normalizing TCRβ template counts to the total amount of DNA usable for TCR sequencing, where the amount of usable DNA was determined by PCR amplification and sequencing of several reference genes that are expected to be present in all nucleated cells. TCR sequences from repertoires were mapped against a set of TCR sequences that are known to react to SARS-CoV-2 by matching on V gene, amino acid sequence and J gene. In brief, these sequences were first identified by Multiplex Identification of T-cell Receptor Antigen Specificity (MIRA)^21^. TCRs that react were further screened for enrichment in COVID-19-positive repertoires collected as part of ImmuneCODE compared to COVID-19-negative repertoires to remove TCRs that may be highly public or cross-reactive to common antigens. Individual response could be quantified by the number and/or frequency of SARS-CoV-2 TCRs seen post-vaccine. TCRs were further analysed at the level specific ORF or position within ORF based on the MIRA antigens. The breadth summary metric is calculated as the number of unique annotated rearrangements out of the total number of unique productive rearrangements, while depth summary metric corresponds to the sum frequency of those rearrangements in the repertoire. Sequences of known variants were obtained from GISAID (www.gisaid.org) and aligned to known MIRA antigen locations.

### Reporting summary

Further information on research design is available in the Nature Research Reporting Summary linked to this paper.

## Online content

Any methods, additional references, Nature Research reporting summaries, source data, extended data, supplementary information, acknowledgements, peer review information; details of author contributions and competing interests; and statements of data and code availability are available at 10.1038/s41586-021-03681-2.

### Supplementary information

Reporting Summary

## Figures and Tables

**Extended Data Table 1:**
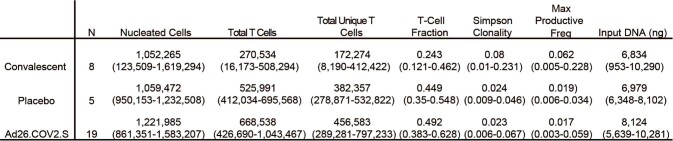
TCRβ repertoire analysis

Cell parameters analysed for TCRβ sequencing. *n* = 32 independent samples (8 SARS-CoV-2 convalescent individuals, 5 placebo recipients, 19 Ad26.COV2.S vaccine recipients).

## Data Availability

All data are available in the manuscript and Supplementary information.
