# Supplementary material for: Immunogenicity of Ad26.COV2.S vaccine against SARS-CoV-2 variants in humans
Source: Nature. 2021 Jun 9;596(7871):268–72. doi: 10.1038/s41586-021-03681-2 (PMC8357629; doi:10.1038/s41586-021-03681-2)
Supplement: Supplementary file 1 — Reporting Summary [file 41586_2021_3681_MOESM1_ESM.pdf]

nature research

Corresponding author(s): Dan H. Barouch

Last updated by author(s): May 22, 2021

Reporting Summary

Nature Research wishes to improve the reproducibility of the work that we publish. This form provides structure for consistency and transparency in reporting. For further information on Nature Research policies, see our Editorial Policies and the Editorial Policy Checklist.

Statistics

For all statistical analyses, confirm that the following items are present in the figure legend, table legend, main text, or Methods section.

n/a	Confirmed	
☐	☒	The exact sample size (n) for each experimental group/condition, given as a discrete number and unit of measurement
☐	☒	A statement on whether measurements were taken from distinct samples or whether the same sample was measured repeatedly
☐	☒	The statistical test(s) used AND whether they are one- or two-sided<br>*Only common tests should be described solely by name; describe more complex techniques in the Methods section.*
☐	☒	A description of all covariates tested
☐	☒	A description of any assumptions or corrections, such as tests of normality and adjustment for multiple comparisons
☐	☒	A full description of the statistical parameters including central tendency (e.g. means) or other basic estimates (e.g. regression coefficient) AND variation (e.g. standard deviation) or associated estimates of uncertainty (e.g. confidence intervals)
☒	☐	For null hypothesis testing, the test statistic (e.g. F, t, r) with confidence intervals, effect sizes, degrees of freedom and P value noted<br>*Give P values as exact values whenever suitable.*
☒	☐	For Bayesian analysis, information on the choice of priors and Markov chain Monte Carlo settings
☒	☐	For hierarchical and complex designs, identification of the appropriate level for tests and full reporting of outcomes
☒	☐	Estimates of effect sizes (e.g. Cohen's d, Pearson's r), indicating how they were calculated

Our web collection on statistics for biologists contains articles on many of the points above.

Software and code

Policy information about availability of computer code

Data collection	None
Data analysis	Analysis of data was performed using FlowJo v9.9 and GraphPad Prism 8.4.2 (GraphPad Software).

For manuscripts utilizing custom algorithms or software that are central to the research but not yet described in published literature, software must be made available to editors and reviewers. We strongly encourage code deposition in a community repository (e.g. GitHub). See the Nature Research guidelines for submitting code & software for further information.

Data

Policy information about availability of data

All manuscripts must include a data availability statement. This statement should provide the following information, where applicable:

- Accession codes, unique identifiers, or web links for publicly available datasets
- A list of figures that have associated raw data
- A description of any restrictions on data availability

Data availability statement included. All data are available in the manuscript and supplementary material.

Field-specific reporting

Please select the one below that is the best fit for your research. If you are not sure, read the appropriate sections before making your selection.

☒ Life sciences ☐ Behavioural & social sciences ☐ Ecological, evolutionary & environmental sciences

For a reference copy of the document with all sections, see nature.com/documents/nr-reporting-summary-flat.pdf

Life sciences study design

All studies must disclose on these points even when the disclosure is negative.

Sample size	Sample size is 25 indivduals (5 individuals/group reflecting 20 vaccine recipients and 5 placebo recipients). This sample size can differentiate large differences in immunogenicity; details provided in the primary clinical trial manuscript (Stephenson et al. JAMA 2021; 325:1535-1544).
Data exclusions	No data were excluded.
Replication	Virologic and immunologic measures were generally performed in duplicate. Technical replicates were minimally different. All attempts at replication were successful.
Randomization	Participants were randomly allocated to groups.
Blinding	The study was double blinded. All immunologic and virologic assays were also performed blinded.

Reporting for specific materials, systems and methods

We require information from authors about some types of materials, experimental systems and methods used in many studies. Here, indicate whether each material, system or method listed is relevant to your study. If you are not sure if a list item applies to your research, read the appropriate section before selecting a response.

Materials & experimental systems

n/a	Involved in the study	
☐	☒	Antibodies
☐	☒	Eukaryotic cell lines
☒	☐	Palaeontology and archaeology
☒	☐	Animals and other organisms
☐	☒	Human research participants
☐	☒	Clinical data
☒	☐	Dual use research of concern

Methods

n/a	Involved in the study	
☒	☐	ChIP-seq
☐	☒	Flow cytometry
☒	☐	MRI-based neuroimaging

Antibodies

Antibodies used	For ELISA and ELISPOT assays anti-macaque IgG HRP (NIH NHP Reagent Program), rabbit polyclonal anti-human IFN-γ (U-Cytech); for ICS assays mAbs from BD against CD279 (clone EH12.1, BB700), CD38 (clone OKT10, PE), CD28 (clone 28.2, PE CY5), CD4 (clone L200, BV510), CD45 (clone D058-1283, BUV615), CD95 (clone DX2, BUV737), CD8 (clone SK1, BUV805), Ki67 (clone B56, FITC), CD69 (clone TP1.55.3, ECD), IL10 (clone JES3-9D7, PE CY7), IL13 (clone JES10-5A2, BV421), TNF-α (clone Mab11, BV650), IL4 (clone MP4-25D2, BV711), IFN-γ (clone B27; BUV395), IL2 (clone MQ1-17H12, APC), CD3 (clone SP34.2, Alexa 700) (BD); for 800CW-conjugated goat-anti-human secondary antibody (Li-COR); anti-rhesus IgG1, IgG2, IgG3, IgA, IgM (NIH NHP Reagent Program); tertiary goat anti-mouse IgG-PE antibody (Southern Biotech), anti-CD107a (PE-Cy7, BD), anti-CD56 (PE-Cy7, BD), anti-MIP-1β (PE, BD), mouse anti-human IFN-γ monoclonal antibody (BD), Streptavidin-alkaline phosphatase antibody (Southern Biotech), anti-human IgG HRP (Invitrogen). Antibodies were used at manufacturer's concentrations and were titrated prior to use.
Validation	mAbs used according to manufacturer's instructions and previously published methods; mAbs were titrated with human PBMC and assessed for specificity prior to use

Eukaryotic cell lines

Policy information about cell lines

Cell line source(s)	HEK293T from ATCC
Authentication	Commerically purchased (ATCC) and evaluated in control experiments prior to use
Mycoplasma contamination	Negative for mycoplasma

Commonly misidentified lines (See ICLAC register)	None were utilized

Human research participants

Policy information about studies involving human research participants

Population characteristics	Healthy adults 18-55, mixed gender; details provided in the primary clinical trial manuscript (Stephenson et al. JAMA 2021; 325:1535-1544)
Recruitment	Participants were recruited by education and outreach programs; details provided in the primary clinical trial manuscript (Stephenson et al. JAMA 2021; 325:1535-1544)
Ethics oversight	BIDMC institutional review board; details provided in the primary clinical trial manuscript (Stephenson et al. JAMA 2021; 325:1535-1544)

Note that full information on the approval of the study protocol must also be provided in the manuscript.

Clinical data

Policy information about clinical studies

All manuscripts should comply with the ICMJE guidelines for publication of clinical research and a completed CONSORT checklist must be included with all submissions.

Clinical trial registration	NCT04436276
Study protocol	Primary clinical trial and protocol published (Stephenson et al. JAMA 2021; 325:1535-1544)
Data collection	25 participants were enrolled from July 29, 2020 to August 7, 2020, and the follow-up for this day 71 interim analysis was completed on October 3, 2020. This study was conducted at a single clinical site in Boston, MA as part of a randomized, double-blinded, placebo-controlled phase 1/2a clinical trial (COV1001) of Ad26.COV2.S.
Outcomes	Variant immunogenicity data are reported in this manuscript. Safety and immunogenicity are reported in the primary clinical trial manuscript (Stephenson et al. JAMA 2021; 325:1535-1544)

Flow Cytometry

Plots

Confirm that:

- [x] The axis labels state the marker and fluorochrome used (e.g. CD4-FITC).
- [x] The axis scales are clearly visible. Include numbers along axes only for bottom left plot of group (a 'group' is an analysis of identical markers).
- [x] All plots are contour plots with outliers or pseudocolor plots.
- [x] A numerical value for number of cells or percentage (with statistics) is provided.

Methodology

Sample preparation	10^6 PBMCs/well were re-suspended in 100 µL of R10 media
Instrument	BD FACSymphony
Software	FlowJo v10
Cell population abundance	See Extended Data Fig. 3
Gating strategy	See Extended Data Fig. 3; FSC/SSC gating of the starting cell population, live/dead staining, gating on CD3+CD4+ or CD3+CD8+ cell populations

- [x] Tick this box to confirm that a figure exemplifying the gating strategy is provided in the Supplementary Information.
